# Troponin and BNP are markers for subsequent non-ischaemic congestive heart failure: the Caerphilly Prospective Study (CaPS)

**DOI:** 10.1136/openhrt-2017-000692

**Published:** 2018-02-23

**Authors:** Christopher C Patterson, Stefan Blankenberg, Yoav Ben-Shlomo, Luke Heslop, Anthony Bayer, Gordon Lowe, Tanja Zeller, John Gallacher, Ian Young, John W G Yarnell

**Affiliations:** 1 Centre for Public Health, Queen’s University Belfast, Belfast, UK; 2 Department of General and Interventional Cardiology, University Heart Centre Hamburg, Hamburg, Germany; 3 DZHK German Center for Cardiovascular Research, Partner Sites Hamburg, Lubeck, Kiel, Hamburg, Germany; 4 School of Social and Community Medicine, University of Bristol, Bristol, UK; 5 Division of Population Medicine, Cardiff University School of Medicine, Cardiff, UK; 6 Institute of Cardiovascular and Medical Sciences, University of Glasgow, Glasgow, UK; 7 Department of Psychiatry, University of Oxford, Warneford Hospital, Oxford, UK

**Keywords:** heart failure, epidemiology, stroke, atherosclerosis

## Abstract

**Objective:**

To examine the long-term predictive value of 28 biomarkers for subsequent non-ischaemic congestive heart failure (CHF) and separately for other cardiovascular outcomes (myocardial infarction (MI) and stroke).

**Methods:**

The Caerphilly Prospective Study recruited 2171 men aged 55–69 years from the general population in 1989–1993; men were screened for evidence of cardiovascular disease (CVD) and followed for clinical cardiovascular events. Fasting blood samples were stored at −70°C until assayed for novel biomarkers in 2010–2013. A competing risks proportional hazards regression analysis was used to estimate subhazard ratios (SHRs) for each biomarker for each cardiovascular outcome.

**Results:**

During follow-up (average 13 years), only new, initial events were evaluated in the whole cohort: 584 MIs, 313 strokes and 261 episodes of CHF (not associated with acute MI). In a subcohort of men who had no clinical history or evidence of CVD at baseline examination (n=1279) those in the top third of the distributions of troponin and B-type natriuretic peptide (BNP) showed a threefold increase in risk for subsequent CHF as a *first* event after adjustment for all conventional risk factors (SHRs 3.37, 95% CI 1.39 to 8.14 and 3.23, 95% CI 1.45 to 7.23), respectively, in contrast to moderate elevations in risk for acute MI (troponin SHR 1.63, 95% CI 1.10 to 2.41) and for stroke (BNP SHR 1.75 95% CI 1.06 to 2.88).

**Conclusion:**

Troponin and BNP could be considered as potentially useful screening tools to detect subjects without prior CVD at increased risk of developing CHF in subsequent years in addition to having lesser roles for predicting subsequent MI (troponin) or stroke (BNP).

**Key questions**

**What is already known about this subject?**

- Chronic heart failure is a leading cause of mortality and morbidity and certain markers such as B-type natriuretic peptide (BNP) have been shown to help predict subsequent heart failure in subjects without a history of prior cardiovascular disease (CVD).
- Troponin levels have been investigated as a predictor of subsequent CVD risk and have been suggested to improve risk prediction when added to established risk models.

**What does this study add?**

- We have examined the relationship between separate first CVD events (acute myocardial infaction (MI), stroke and chronic congestive heart failure) in a cohort of men with no history or clinical evidence of prior CVD.
- We have examined both the frequency of the individual CVD events and also the utility of risk prediction models for each type of CVD event that included troponin and BNP in addition to established risk factors (also lipoprotein-a for acute MI).

**How might this impact on clinical practice?**

- Our study suggests that both BNP and troponin could usefully help predict subsequent chronic CHF in subjects with no prior evidence of CVD. In our data, troponin and BNP appear to have lesser roles in predicting acute MI and stroke, but these findings should be investigated in larger collaborative studies.

## Introduction

Cardiovascular diseases (CVDs) are the most important cause of death worldwide, projected to account for 24% of deaths by 2030 compared with 12% for cancers.^1^ However, the increase in CVDs is occurring in low-income and middle-income countries, while decreasing in high-income countries.^2^

Ischaemic heart disease (IHD) is the most common manifestation of CVD in high-income countries, while stroke and congestive heart failure (CHF) tend to predominate in many low-income and middle-income countries.^3^ Primary preventive measures in the general population resulting in lower levels of smoking, hypertension and serum cholesterol appear to account for more than 50% of the decline in mortality from IHD and stroke in several high-income countries,^4^ although adverse trends are occurring worldwide in levels of obesity and diabetes,^3^ which are established risk factors for CHF.^5 6^ CHF is considerably under-reported as a cause of death as coding guidelines in the International Classification of Diseases discourage the recording of heart failure as an underlying cause of death,^7^ particularly if other conditions are present such as IHD or (less commonly) hypertension. Evidence from hospital admissions, however, suggests a steep rise in the prevalence of CHF in high-income countries.^8 9^ A meta-analysis of 26 studies showed CHF to be an important risk factor for subsequent stroke.^10^

There is increasing evidence that coagulation and inflammatory factors play an important role in the pathogenesis of CVDs, which appear to be largely initiated and progressed by atherothrombotic mechanisms.^11^ The role of such biomarkers in prediction of CHF has been less studied, but epidemiological studies have suggested a role for inflammatory cytokines with the development of CHF.^12^ Cardiac peptides have become established in clinical practice in the diagnosis and treatment of CHF,^13^ and several epidemiological studies^14^ have suggested B-type natriuretic peptide (BNP), or its inactive N-terminal fragment NT-pro BNP, to be of potential use in risk assessment for subsequent CHF in the general population. We have previously investigated 28 conventional and novel markers representing several pathogenic pathways in relation to CVD and non-CVD mortality.^15^ We now examine if these markers have predictive value for CHF, acute myocardial infarction (MI) and stroke events (fatal and non-fatal) in the Caerphilly Prospective Study (CaPS), a population-based cohort of men examined in 1989–1993.

## Methods

### Study population

CaPS was established in 1979 when men from the general population of the South Wales town of Caerphilly and its surrounding villages were recruited; 89% of the eligible population was examined, and the cohort, which has previously been characterised,^16^ was re-examined at approximately 5 yearly intervals. A detailed medical and lifestyle history was obtained, the London School of Hygiene & Tropical Medicine (LSHTM) chest pain questionnaire was administered, a full resting 12-lead ECG was recorded and height, weight and blood pressure were measured as described previously.^17^ ECGs were coded by two experienced coders according to the Minnesota coding scheme.

This report is based on the second follow-up examination of the men (phase 3) in the period 1989–1993 when the men were predominantly aged 55–69 years. Of the 2171 men examined, a fasting blood sample was obtained subsequently from 1911 (88%) men.

A subsample of men who had no evidence of existing ischaemia, angina, severe chest pain (from the LSHTM questionnaire) or history of stroke at the baseline examination were selected to examine the first subsequent cardiovascular events in this cohort as defined below. Men with any evidence of ECG ischaemia using the Whitehall criteria for ECG ischaemia (Q-waves, ST depression, T wave inversion and left bundle branch block) were excluded from this subsample. All men gave written informed consent.

### Blood collection, storage and analysis

At a separate appointment, shortly after the clinical examination, a venous blood sample was collected from each man after an overnight fast. Fresh samples were used to measure acute phase reactants and routine lipids and routine biochemistry as described previously.^17^ Other aliquots were taken, and plasma or serum were separated within 1 hour and stored at −20°C for up to 6 hours and then at −70°C until laboratory analysis in 2010–2013.

### Biomarker assays

Assays for high-sensitivity troponin, BNP, C reactive protein (CRP), growth differentiation factor-15, cystatin-C, creatinine, ferritin and lipoprotein-a (Lp-a) were performed in the BiomarCaRE laboratory as described elsewhere.^18^ Vascular cell adhesion molecule-1, E-selectin, interleukin-6 (IL-6), pregnancy-associated plasma protein-A, retinol binding protein-4, fetuin-A and IL-6 receptor were assayed using commercial ELISAs, and vitamin B6 was measured using high-performance liquid chromatography. Coefficients of variation for assays in our panel of biomarkers for blindly presented duplicate aliquots varied from 1% to 27% as reported previously.^15^

### Follow-up

#### Fatal events

The men have been followed for mortality through flagging by the Health and Social Care Information Centre with follow-up until 28 February 2012. The underlying cause of death coded from the death certificate was used. MI/IHD codes were International Classification of Diseases, 9th revision (ICD-9) 410–414, ICD, 10th revision (ICD-10) I20–I25, stroke codes were ICD-9 430–438 and ICD-10 I60–I69 and CHF codes were ICD-9 428 and ICD-10 I50.

#### Non-fatal events

We tracked non-fatal events for men in the cohort who gave written consent to link their data to their hospital records in the Hospital Episode Statistics (HES) system as described previously.^17^ In the current report, all hospital admissions for Wales from January 1998 to 28 February 2012 were examined for possible disease episodes. We used the identical ICD-10 codes as for deaths with the addition of I46 for IHD, G45 and G46 for stroke and I11 and I13 for CHF.

Electronic health records from primary care were used to validate diagnoses obtained from HES. These are available for all residents in the Caerphilly area and contain scanned copies of hospital discharge letters dating from the late 1990s. Subjects were also re-examined in 1994–1998 and in 2002–2005 when detailed questionnaires were used to capture details of new clinical events.

Prior to the year 2000, an MI/IHD or stroke event was validated by an independent medical committee using hospital notes and primary care records. Stroke was based on clinical history and CT as described previously.^16^

After the year 2000, a new medical committee (LH, YB-S and AB) reviewed hospital admissions from 1998 to 2012 and summary electronic GP records predating these hospital admissions. A diagnosis of acute MI was based on the consultant discharge letter, diagnostic troponin values and evidence of ischaemia on ECG as in the Third Universal Definition of Myocardial Infarction criteria.^19^ Stroke was classified as transient ischaemic attack, ischaemic stroke, intracerebral haemorrhagic stroke or stroke of uncertain subtype by the committee using clinical records and CT, but possible subarachnoid haemorrhage events were not included. Possible CHF events were diagnosed according to the Framingham criteria^20^ supported by echocardiography when available and/or a diagnosis made by a consultant cardiologist. Acute CHF episodes of limited duration (<24 hours) following MI were excluded from the validated CHF cases. In the subsample of men with no evidence of prior IHD/stroke, as defined above, events defined as first CHF events were considered to be non-ischaemic.

### Statistical methods

Analysis is based on 1112 men with no history of CVD who provided fasting blood samples and had complete data on all relevant covariates. The majority of biomarkers showed skewed distributions, and small numbers of men had results below a lower limit of detection for some biomarker assays. Biomarker results were therefore summarised using median and IQRs and compared between subgroups using the Mann-Whitney U test. Biomarker distributions were divided into thirds by sample tertiles for further analysis.

A competing risk formulation of Cox’s proportional hazards regression model was used to estimate subhazard ratios (SHRs) for each CVD outcome with other outcomes treated as competing risks.^21^ This approach models cumulative incidence functions rather than cause-specific hazard functions.^22^ Tests for linear trend in the SHRs across the thirds of each biomarker were obtained together with tests for deviation from linearity across the thirds. A time-dependent covariate test was used to investigate the subhazard proportionality assumption that specifies that there is no change in the SHRs with time. A significant interaction between the trend in a categorised biomarker and time in the Cox model provided evidence of failure of the proportional subhazards assumption.

Harrell’s C statistic^23^ was calculated as a measure of the predictive ability of regression models that included troponin and BNP separately and together in addition to conventional risk factors.

To reduce the risk of type 1 errors arising in the multiple testing, the 1% significance level was employed for all tests. Analyses were performed using SPSS version 20 and Stata release 12.

## Results

The mean period of follow-up for the 2171 men was 13.0 years (range 0.01–22.5 years) with a total of 28 312 person-years. The numbers of men in various subgroups analysed in this report are listed in online supplementary table 1.

10.1136/openhrt-2017-000692.supp1 Supplementary file 1

**Figure 1:**
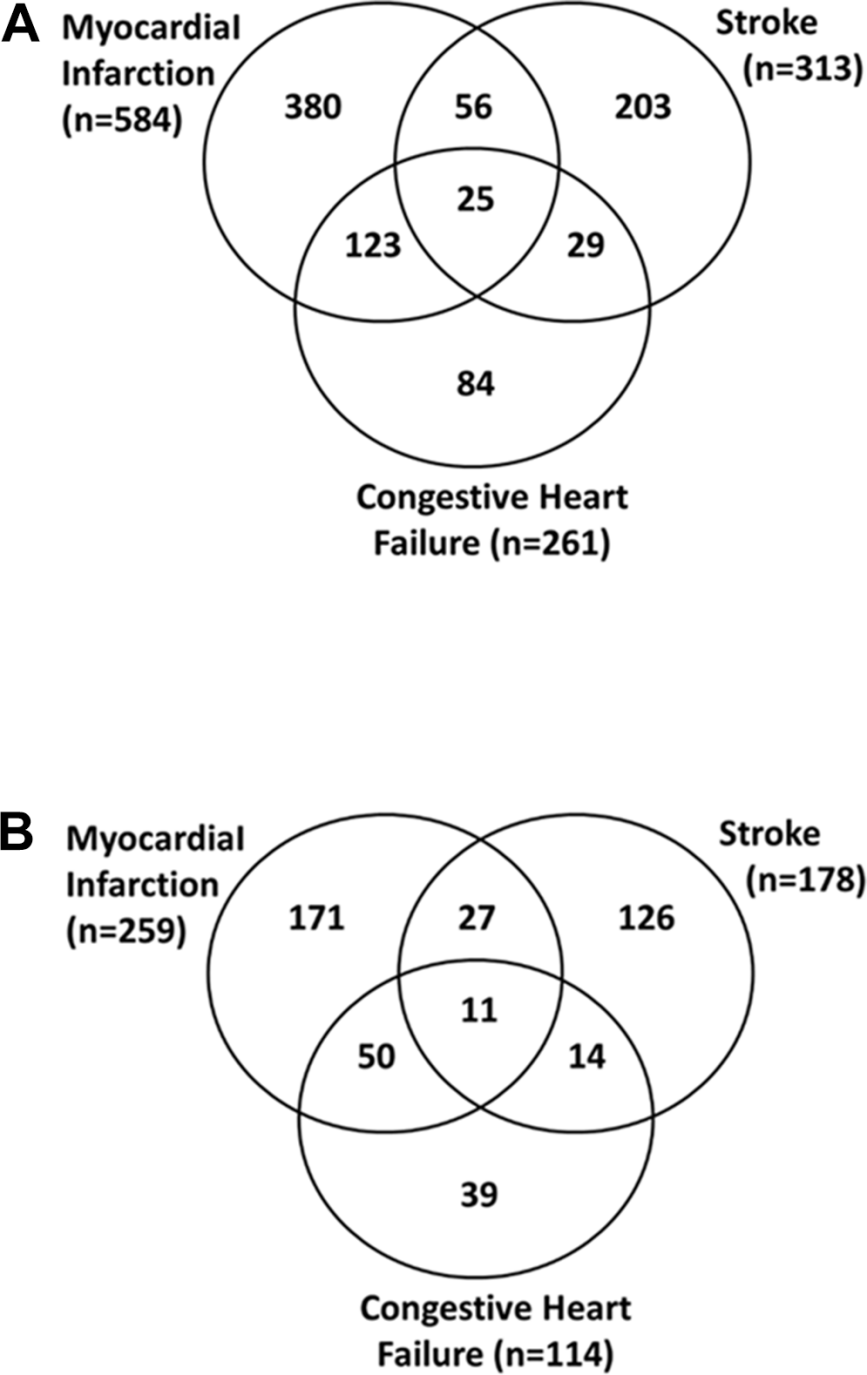
Outcome events in the Caerphilly Prospective Study (CaPS) during 13 years of follow-up. (A) All men (n = 2171), (B) men without pre-existing CVD (n=1279).

Figure 1A shows the number of outcome events in all subjects: 584 acute MI/IHD events (at an average of 9.3 years of follow-up), 313 stroke events (at an average of 9.8 years of follow-up) and 261 CHF events (at an average of 12.0 years of follow-up). However, these events occurred in 900 men as many men had more than one type of outcome event. For example, of the 261 men who experienced a CHF event, 148 (123+25) also had acute MI/IHD and 54 (29+25) a stroke; in 61 of these 148 men, CHF was the first event. When a CHF event occurred during the same day as an MI/IHD or stroke event, the latter was counted as the first event. In the whole cohort, which included men with prior CVD, 84 men experienced a CHF event without any MI/IHD or stroke event. Therefore, of 900 men with any CVD event, 145 (16%) had a CHF event first. However, as our cohort did not exclude men with evidence of prior CVD, as defined in the methods section, 80 had such evidence indicating that 55% of these 145 men who experienced a CHF event first had prior CVD.

In the light of this observation, we investigated the subsample of men (n=1279) who had no evidence of prior CVD, and we categorised first CHF events as non-ischaemic. Figure 1B shows that 114 CHF events occurred in this subcohort, and there were also 259 MI/IHD events and 178 strokes that occurred in a total of 438 men.

The numbers of first events were as follows: 217 for acute MI/IHD, 158 for stroke and 63 for CHF, for a total of 438 first CVD events. In these, 438 men 63 (14%) had a CHF event first. Therefore, in men with no evidence of CVD at baseline, 55% (63/114) of CHF events preceded other cardiovascular outcomes or occurred as the only type of outcome during the follow-up period.

Table 1 shows the median value of each of the biomarkers grouped by pathogenic pathway in men with no event during follow-up, those with a first MI/IHD event, those with a first stroke and in those with a first CHF event. Only 1176 fasting men without evidence of prior CVD at baseline were included in this analysis.

**Table 1:** Median (IQR) values of biomarkers in 1176 fasting men with no CVD at entry by status defined by type of first event occurring during follow-up

Biomarker	No event during follow-up		New MI/IHD event during follow-up			New stroke event during follow-up			New CHF event during follow-up		
	n	Median (IQR)	n	Median (IQR)	P	n	Median (IQR)	P	n	Median (IQR)	P
Cardiac and vascular											
Troponin (pg/mL)	751	5.4 (4.3–6.8)	186	6.3 (5.1–7.6)	**<0.001**	125	5.4 (4.6–7.3)	0.37	51	6.9 (5.2–9.6)	**<0.001**
BNP (pg/mL)	743	19.9 (12.8–30.2)	184	21.6 (13.1–35.4)	0.13	124	23.8 (15.9–41.2)	**0.002**	53	35.5 (20.5–65.0)	**<0.001**
Inflammatory											
CRP (mg/L)	748	2.05 (1.07–4.13)	188	2.56 (1.49–4.54)	**0.01**	127	2.31 (1.25–4.05)	0.19	51	2.75 (1.57–4.69)	0.10
IL-6 (pg/mL)	750	2.04 (1.42–3.22)	188	2.07 (1.49–3.27)	0.33	127	2.31 (1.63–3.07)	0.11	51	2.21 (1.56–3.24)	0.29
IL-6 receptor (ng/mL)	751	37.8 (30.5–44.9)	188	38.0 (30.8–46.9)	0.55	126	37.7 (29.8–45.0)	0.61	51	38.0 (28.8–43.7)	0.62
Lipids											
Cholesterol (mmol/L)	781	6.0 (5.3–6.8)	195	6.5 (6.0–7.2)	**<0.001**	137	6.0 (5.4–6.6)	0.77	54	6.2 (5.5–6.9)	0.50
Triglyceride (mmol/L)	781	1.5 (1.1–2.2)	195	1.7 (1.3–2.4)	**<0.001**	137	1.5 (1.1–2.2)	0.83	54	1.6 (1.0–2.0)	0.37
Lp-a (mg/dL)	743	7.5 (3.5–18.5)	188	11.4 (5.1–30.8)	**0.001**	127	7.3 (2.6–30.6)	0.97	51	6.6 (2.4–20.1)	0.42
Adhesion molecules											
VCAM-1 (ng/mL)	750	1282 (1153–1437)	188	1272 (1106–1463)	0.68	127	1317 (1162–1454)	0.35	51	1322 (1216–1497)	0.08
E-selectin (ng/mL)	751	25.8 (19.0–33.4)	188	25.4 (19.0–34.8)	0.91	127	27.9 (21.3–37.0)	0.04	51	30.2 (20.6–38.6)	0.16
Liver function											
Alkaline phosphatase (IU/L)	776	85 (70–102)	194	86 (71–103)	0.42	137	92 (74–111)	**0.003**	54	87 (70–105)	0.60
Aspartate transaminase (IU/L)	773	22 (18–27)	192	21 (18–25)	0.07	137	20 (17–25)	**0.001**	53	24 (19–29)	0.22
Alanine transaminase (IU/L)	777	22 (17–29)	193	23 (16–28)	0.86	137	21 (17–26)	0.40	54	23 (18–29)	0.45
Gamma-GT (IU/L)	781	27 (20–42)	195	30 (21–44)	0.13	137	28 (20–40)	0.97	54	28 (23–38)	0.41
Glutamate dehydrogenase (IU/L)	362	2.0 (1.2–3.3)	102	2.2 (1.2–3.7)	0.57	65	2.0 (1.0–3.3)	0.94	25	2.3 (1.0–3.4)	0.99
Acute phase											
White cell count (×10^9^/L)	779	5.92 (5.07–7.07)	196	6.41 (5.30–7.43)	**0.006**	137	6.20 (5.13–7.70)	0.07	54	6.30 (4.86–7.26)	0.50
Viscosity (mPa·s)	776	167 (162–173)	195	170 (164–176)	**<0.001**	137	169 (163–176)	**0.008**	54	170 (164–180)	0.03
Fibrinogen (g/L)	771	390 (340–450)	195	410 (360–480)	**<0.001**	136	410 (370–470)	0.02	54	400 (340–470)	0.39
Ferritin (ng/mL)	750	112 (64–204)	187	113 (73–171)	0.94	126	1166 (67–184)	0.97	51	136 (70–216)	0.63
Leucocyte activation											
GDF-15 (pg/mL)	751	684 (521–876)	188	717 (571–916)	0.10	127	708 (566–931)	0.07	51	742 (607–910)	0.10
PAPP-A (ng/mL)	749	7.12 (5.12–9.26)	188	7.27 (5.23–9.32)	0.75	127	7.65 (5.88–9.68)	0.05	51	7.80 (6.22–9.40)	0.08
Renal function											
Cystatin-C (mg/L)	748	0.83 (0.75–0.92)	188	0.85 (0.77–0.92)	0.12	127	0.85 (0.78–0.93)	0.05	51	0.87 (0.77–0.96)	0.15
Creatinine (mg/dL)	775	97 (88–106)	194	97 (88–106))	0.49	137	97 (88–106))	0.40	53	97 (88–106)	0.53
Insulin resisitance											
Glucose (mmol/L)	777	5.2 (4.9–5.6)	195	5.4 (5.0–5.9)	0.02	137	5.4 (5.0–6.0)	**0.005**	54	5.5 (5.0–5.8)	0.09
Retinol-binding protein (mg/L)	750	31.6 (26.0–37.9)	188	31.9 (26.1–37.9)	1.00	126	29.2 (24.8–34.9)	0.04	51	29.4 (24.6–36.0)	0.06
Fetuin-A (mg/L)	749	256 (218–306)	188	273 (224–321)	0.16	126	266 (226–308)	0.30	51	265 (236–298)	0.51
Others											
Uric acid (mg/L)	780	33 (29–39)	195	33 (29–38)	0.89	137	33 (28–38)	0.43	54	35 (30–39)	0.79
Vitamin B6 (nmol/L)	750	40.9 (30.4–56.0)	184	40.3 (29.0–54.5)	0.41	122	37.2 (27.3–50.6)	0.05	54	37.8 (29.4–51.1)	0.42

P values are for comparisons with the no event group.

BNP, B-type natriuretic peptide; CHF, congestive heart failure; CRP, C reative protein; CVD, cardiovascular disease; GDF-15, growth differentiation factor-15; IHD, ischaemic heart disease; IL, interleukin; Lp-a, lipoprotein-a; MI, myocardial infarction; PAPP-A, pregnancy-associated plasma protein-A; VCAM-1, vascular cell adhesion molecule-1.

Men who experienced a first MI/IHD or a first CHF event (but not men who experienced a first stroke event) showed significantly higher levels of troponin than those men with no events. BNP levels were significantly higher in men with a first stroke and markedly and significantly higher in men with a first CHF event.

One inflammatory marker, CRP, and three acute phase proteins, white blood cell count, viscosity and fibrinogen tended to show higher levels in men with all types of new CVD events, but only men with new first MI/IHD events (the most frequent CVD outcome) showed significant differences from men with no events. Lipid levels, total cholesterol, triglycerides and Lp-a were notably higher in men with new MI/IHD. Glucose levels were higher in men with all types of new CVD events compared with men with no events, although the result attained significance only for stroke outcome. Alkaline phosphatase showed significantly raised levels in new stroke subjects, while aspartate transaminase levels were significantly lower in this group.

In table 2, the distributions of each biomarker have been divided into thirds and crude (adjusted for age only) and fully adjusted SHRs have been calculated. Adjustment was made for the following conventional risk factors: age, smoking, diabetes, systolic blood pressure, total cholesterol, total triglycerides, body mass index and male/female family history of premature CHD.

**Table 2:** Sub-hazard ratios for first event after entry by thirds of biomarkers in a competing risk model for 1112 fasting men with no CVD at entry and complete data

		MI/IHD			Stroke			Congestive heart failure		
		Sub-hazard ratio (95% CI)			Sub-hazard ratio (95% CI)			Sub-hazard ratio95% CI)		
	n	Events	Crude*	Adjusted†	Events	Crude*	Adjusted†	Events	Crude*	Adjusted†
Troponin										
Low <4.9 pg/mL	358	40	1.00‡	1.00‡	42	1.00	1.00	7	1.00‡	1.00‡
Mid 4.9–6.4 pg/mL	341	50	1.35 (0.89 to 2.05)	1.09 (0.71 to 1.67)	39	0.90 (0.58 to 1.38)	0.93 (0.58 to 1.49)	12	1.73 (0.70 to 4.30)	1.80 (0.70 to 4.60)
High >6.4 pg/mL	360	84	2.22 (1.53 to 3.22)	1.63 (1.10 to 2.41)	35	0.73 (0.46 to 1.16)	0.68 (0.41 to 1.13)	27	3.68 (1.63 to 8.30)	3.37 (1.39 to 8.14)
BNP										
Low <15.8 pg/mL	348	54	1.00	1.00	26	1.00	1.00	8	1.00 ‡	1.00 ‡
Mid 15.8–27.8 pg/mL	345	53	0.99 (0.67 to 1.45)	0.95 (0.64 to 1.40)	36	1.27 (0.75 to 2.13)	1.26 (0.74 to 2.15)	11	1.31 (0.52 to 3.30)	1.26 (0.50 to 3.15)
High >27.8 pg/mL	355	66	1.21 (0.84 to 1.73)	1.20 (0.83 to 1.73)	53	1.81 (1.11 to 2.94)	1.75 (1.06 to 2.88)	29	3.36 (1.50 to 7.52)	3.23 (1.45 to 7.23)
CRP										
Low <1.49 mg/L	349	42	1.00	1.00	35	1.00	1.00	10	1.00	1.00
Mid 1.49–3.32 mg/L	354	65	1.54 (1.04 to 2.27)	1.28 (0.86 to 1.92)	41	1.14 (0.72 to 1.79)	1.11 (0.70 to 1.75)	20	1.94 (0.91 to 4.12)	1.62 (0.74 to 3.56)
High >3.32 mg/L	356	69	1.62 (1.10 to 2.40)	1.25 (0.83 to 1.91)	41	1.06 (0.67 to 1.68)	1.03 (0.64 to 1.68)	16	1.47 (0.66 to 3.26)	1.10 (0.49 to 2.46)
IL-6										
Low <1.64 pg/mL	351	53	1.00	1.00	30	1.00	1.00	14	1.00	1.00
Mid 1.64–2.76 pg/mL	356	65	1.22 (0.85 to 1.76)	1.04 (0.71 to 1.52)	42	1.36 (0.85 to 2.17)	1.35 (0.85 to 2.13)	18	1.23 (0.61 to 2.46)	1.17 (0.57 to 2.40)
High >2.76 pg/mL	354	58	1.07 (0.73 to 1.57)	0.89 (0.60 to 1.32)	45	1.39 (0.87 to 2.23)	1.32 (0.82 to 2.11)	14	0.89 (0.41 to 1.92)	0.71 (0.31 to 1.61)
IL-6 receptor										
Low <32.9 ng/mL	353	58	1.00	1.00	45	1.00	1.00	14	1.00	1.00
Mid 32.9–42.3 ng/mL	351	54	0.93 (0.65 to 1.35)	0.90 (0.62 to 1.32)	31	0.69 (0.43 to 1.09)	0.68 (0.42 to 1.10)	16	1.15 (0.56 to 2.36)	1.23 (0.60 to 2.51)
High >42.3 ng/mL	357	64	1.09 (0.77 to 1.56)	1.03 (0.71 to 1.48)	40	0.84 (0.55 to 1.29)	0.85 (0.55 to 1.32)	16	1.10 (0.53 to 2.30)	1.09 (0.53 to 2.26)
Cholesterol										
Low <5.7 mmol/L	363	35	1.00‡	1.00‡	42	1.00	1.00	17	1.00	1.00
Mid 5.7–6.5 mmol/L	358	59	1.79 (1.18 to 2.72)	1.77 (1.15 to 2.70)	49	1.21 (0.80 to 1.83)	1.21 (0.80 to 1.84)	15	0.91 (0.45 to 1.81)	1.04 (0.51 to 2.14)
High >6.5 mmol/L	391	90	2.62 (1.77 to 3.87)	2.48 (1.63 to 3.77)	36	0.80 (0.51 to 1.24)	0.78 (0.49 to 1.23)	18	1.00 (0.51 to 1.93)	1.27 (0.62 to 2.61)
Triglyceride										
Low <1.3 mmol/L	362	46	1.00‡	1.00	39	1.00	1.00	17	1.00	1.00
Mid 1.3–1.9 mmol/L	373	63	1.36 (0.93 to 1.98)	1.09 (0.73 to 1.62)	49	1.26 (0.83 to 1.91)	1.31 (0.84 to 2.05)	20	1.17 (0.61 to 2.23)	0.93 (0.49 to 1.77)
High >1.9 mmol/L	377	75	1.67 (1.16 to 2.42)	1.17 (0.77 to 1.78)	39	1.03 (0.66 to 1.60)	1.03 (0.65 to 1.63)	13	0.77 (0.38 to 1.56)	0.59 (0.28 to 1.26)
Lp-a										
Low <5.0 mg/dL	354	45	1.00‡	1.00‡	46	1.00	1.00	22	1.00	1.00
Mid 5.0–13.6 mg/dL	350	55	1.26 (0.85 to 1.86)	1.39 (0.93 to 2.09)	36	0.78 (0.50 to 1.20)	0.80 (0.52 to 1.24)	11	0.49 (0.24 to 1.01)	0.41 (0.19 to 0.86)
High >13.6 mg/dL	350	76	1.81 (1.25 to 2.61)	1.84 (1.26 to 2.69)	35	0.76 (0.49 to 1.19)	0.81 (0.52 to 1.27)	13	0.59 (0.30 to 1.18)	0.52 (0.25 to 1.08)
VCAM-1										
Low <1195 ng/mL	349	69	1.00	1.00	32	1.00	1.00	10	1.00	1.00
Mid 1195–1379 ng/mL	357	49	0.66 (0.46 to 0.95)	0.64 (0.44 to 0.93)	43	1.29 (0.82 to 2.04)	1.30 (0.81 to 2.06)	19	1.82 (0.84 to 3.93)	1.84 (0.86 to 3.94)
High >1379 ng/mL	355	58	0.78 (0.55 to 1.11)	0.84 (0.59 to 1.20)	42	1.21 (0.76 to 1.91)	1.12 (0.70 to 1.80)	17	1.56 (0.70 to 3.46)	1.76 (0.78 to 3.97)
E-selectin										
Low <21.8 ng/mL	358	64	1.00	1.00	33	1.00	1.00	14	1.00	1.00
Mid 21.8–31.0 ng/mL	355	55	0.85 (0.59 to 1.23)	0.79 (0.55 to 1.15)	40	1.28 (0.81 to 2.04)	1.26 (0.79 to 2.02)	13	0.96 (0.45 to 2.04)	0.89 (0.42 to 1.90)
High >31.0 ng/mL	349	57	0.90 (0.63 to 1.28)	0.78 (0.53 to 1.13)	44	1.49 (0.95 to 2.34)	1.38 (0.87 to 2.19)	19	1.48 (0.75 to 2.93)	1.32 (0.65 to 2.68)
Alkaline phosphatase										
Low <76 IU/L	384	61	1.00	1.00	33	1.00	1.00	18	1.00	1.00
Mid 76–96 IU/L	369	63	1.05 (0.74 to 1.50)	0.93 (0.65 to 1.34)	44	1.36 (0.87 to 2.14)	1.42 (0.90 to 2.25)	16	0.91 (0.47 to 1.80)	0.84 (0.42 to 1.68)
High >96 IU/L	352	59	1.04 (0.72 to 1.48)	0.85 (0.58 to 1.24)	50	1.57 (1.00 to 2.47)	1.50 (0.95 to 2.38)	16	0.90 (0.45 to 1.81)	0.87 (0.43 to 1.76)
Aspartate transaminase										
Low <20 IU/L	380	74	1.00	1.00	61	1.00	1.00	14	1.00	1.00
Mid 20–24 IU/L	347	60	0.88 (0.62 to 1.23)	0.96 (0.68 to 1.35)	29	0.50 (0.32 to 0.78)	0.54 (0.34 to 0.85)	14	1.11 (0.53 to 2.34)	1.04 (0.48 to 2.29)
High >24 IU/L	372	47	0.63 (0.44 to 0.91)	0.68 (0.46 to 0.99)	37	0.62 (0.41 to 0.93)	0.63 (0.41 to 0.96)	21	1.60 (0.82 to 3.15)	1.63 (0.82 to 3.22)
Alanine transaminase										
Low <19 IU/L	364	59	1.00	1.00	39	1.00	1.00	17	1.00	1.00
Mid 19–25 IU/L	382	64	1.05 (0.73 to 1.50)	0.98 (0.68 to 1.42)	54	1.43 (0.94 to 2.17)	1.44 (0.92 to 2.25)	17	1.01 (0.51 to 1.99)	0.91 (0.45 to 1.82)
High >25 IU/L	360	59	1.02 (0.71 to 1.47)	0.93 (0.62 to 1.39)	34	0.99 (0.62 to 1.59)	0.96 (0.57 to 1.62)	16	1.07 (0.54 to 2.13)	1.00 (0.49 to 2.02)
Gamma-GT										
Low <23 IU/L	354	56	1.00	1.00	40	1.00	1.00	12	1.00	1.00
Mid 23–35 IU/L	378	61	1.02 (0.71 to 1.47)	0.88 (0.61 to 1.28)	44	1.04 (0.68 to 1.59)	1.04 (0.67 to 1.61)	24	1.95 (0.98 to 3.88)	1.79 (0.86 to 3.71)
High >35 IU/L	380	67	1.13 (0.79 to 1.61)	0.80 (0.54 to 1.18)	43	1.05 (0.68 to 1.62)	0.99 (0.62 to 1.58)	14	1.14 (0.53 to 2.42)	0.97 (0.45 to 2.13)
Glutamate dehydrogenase										
Low <1.41 IU/L	179	35	1.00	1.00	21	1.00	1.00	7	1.00	1.00
Mid 1.41–2.80 IU/L	175	28	0.82 (0.50 to 1.34)	0.81 (0.48 to 1.35)	17	0.84 (0.44 to 1.60)	0.88 (0.46 to 1.68)	8	1.20 (0.43 to 3.35)	1.25 (0.41 to 3.78)
High >2.80 IU/L	179	36	1.07 (0.68 to 1.70)	1.03 (0.63 to 1.68)	22	1.11 (0.61 to 2.03)	1.15 (0.62 to 2.15)	8	1.23 (0.43 to 3.49)	1.23 (0.43 to 3.56)
White cell count										
Low <5.40×10^9^/L	367	49	1.00^†^	1.00	38	1.00	1.00	17	1.00	1.00
Mid 5.40–6.79×10^9^/L	369	57	1.18 (0.81 to 1.73)	1.13 (0.77 to 1.64)	37	0.98 (0.62 to 1.53)	0.95 (0.60 to 1.49)	12	0.70 (0.34 to 1.47)	0.75 (0.35 to 1.58)
High >6.79×10^9^/L	369	78	1.65 (1.16 to 2.36)	1.44 (1.00 to 2.09)	51	1.37 (0.90 to 2.08)	1.43 (0.90 to 2.27)	20	1.18 (0.62 to 2.26)	1.30 (0.61 to 2.74)
Viscosity										
Low <1.65 mPa.s	388	46	1.00‡	1.00	39	1.00	1.00	14	1.00	1.00
Mid 1.65–1.71 mPa.s	335	62	1.58 (1.08 to 2.31)	1.41 (0.95 to 2.08)	34	0.97 (0.61 to 1.53)	0.98 (0.61 to 1.58)	13	1.03 (0.48 to 2.20)	1.02 (0.48 to 2.17)
High >1.71 mPa.s	378	75	1.70 (1.17 to 2.46)	1.29 (0.87 to 1.92)	53	1.28 (0.85 to 1.95)	1.25 (0.81 to 1.94)	22	1.48 (0.74 to 2.96)	1.44 (0.72 to 2.91)
Fibrinogen										
Low <3.6 g/L	359	50	1.00	1.00	32	1.00	1.00	18	1.00	1.00
Mid 3.6–4.3 g/L	367	60	1.17 (0.81 to 1.70)	1.07 (0.74 to 1.57)	49	1.45 (0.93 to 2.27)	1.57 (0.98 to 2.50)	13	0.67 (0.33 to 1.36)	0.60 (0.29 to 1.26)
High >4.3 g/L	369	73	1.46 (1.02 to 2.10)	1.32 (0.92 to 1.91)	44	1.22 (0.77 to 1.93)	1.27 (0.79 to 2.06)	18	0.89 (0.47 to 1.71)	0.90 (0.46 to 1.74)
Ferritin										
Low <83 ng/mL	355	54	1.00	1.00	37	1.00	1.00	13	1.00	1.00
Mid 83–161 ng/mL	352	67	1.30 (0.91 to 1.85)	1.18 (0.82 to 1.69)	40	1.12 (0.71 to 1.75)	1.13 (0.71 to 1.78)	12	0.95 (0.43 to 2.08)	0.86 (0.38 to 1.91)
High >161 ng/mL	352	54	1.02 (0.70 to 1.49)	0.89 (0.60 to 1.30)	39	1.09 (0.69 to 1.71)	1.04 (0.65 to 1.67)	21	1.70 (0.84 to 3.44)	1.66 (0.78 to 3.51)
GDF-15										
Low <591 pg/mL	358	53	1.00	1.00	35	1.00	1.00	12	1.00	1.00
Mid 591–813 pg/mL	350	63	1.18 (0.81 to 1.71)	1.03 (0.70 to 1.51)	40	1.05 (0.66 to 1.66)	1.00 (0.63 to 1.59)	15	1.19 (0.55 to 2.56)	1.10 (0.49 to 2.45)
High >813 pg/mL	354	60	1.10 (0.75 to 1.61)	0.91 (0.60 to 1.38)	42	1.01 (0.63 to 1.62)	0.89 (0.54 to 1.47)	19	1.39 (0.62 to 3.08)	1.18 (0.50 to 2.78)
PAPP-A										
Low <6.00 ng/mL	353	61	1.00	1.00	32	1.00	1.00	9	1.00	1.00
Mid 6.00–8.52 ng/mL	351	57	0.90 (0.62 to 1.29)	0.91 (0.63 to 1.32)	40	1.23 (0.77 to 1.95)	1.23 (0.76 to 1.98)	20	2.17 (0.99 to 4.74)	2.39 (1.08 to 5.30)
High >8.52 ng/mL	356	58	0.89 (0.62 to 1.27)	1.04 (0.72 to 1.50)	45	1.32 (0.84 to 2.08)	1.31 (0.82 to 2.11)	17	1.78 (0.80 to 3.96)	2.19 (0.97 to 4.94)
Cystatin										
Low <0.79 mg/L	357	53	1.00	1.00	28	1.00	1.00	16	1.00	1.00
Mid 0.79–0.89 mg/L	340	54	1.06 (0.72 to 1.55)	1.06 (0.71 to 1.56)	45	1.62 (1.01 to 2.59)	1.64 (1.02 to 2.64)	10	0.61 (0.28 to 1.35)	0.53 (0.24 to 1.16)
High >0.89 mg/L	362	69	1.26 (0.87 to 1.82)	1.02 (0.69 to 1.52)	44	1.37 (0.84 to 2.24)	1.36 (0.80 to 2.29)	20	1.09 (0.52 to 2.24)	0.99 (0.48 to 2.04)
Creatinine										
Low <0.90 mg/dL	353	72	1.00	1.00	43	1.00	1.00	14	1.00	1.00
Mid 0.90–0.99 mg/dL	363	53	0.71 (0.50 to 1.01)	0.73 (0.51 to 1.04)	42	0.95 (0.62 to 1.45)	0.96 (0.63 to 1.47)	14	0.97 (0.46 to 2.03)	1.00 (0.46 to 2.17)
High >0.99 mg/dL	339	51	0.73 (0.51 to 1.04)	0.73 (0.50 to 1.05)	32	0.74 (0.47 to 1.18)	0.78 (0.49 to 1.26)	18	1.30 (0.65 to 2.63)	1.44 (0.66 to 3.14)
Glucose										
Low <5.1 mmol/L	369	58	1.00	1.00	37	1.00	1.00	14	1.00	1.00
Mid 5.1–5.5 mmol/L	373	55	0.94 (0.65 to 1.36)	0.92 (0.63 to 1.34)	39	1.05 (0.67 to 1.65)	1.04 (0.67 to 1.64)	17	1.22 (0.60 to 2.48)	1.25 (0.61 to 2.57)
High >5.5 mmol/L	363	71	1.26 (0.89 to 1.79)	1.19 (0.80 to 1.76)	51	1.43 (0.93 to 2.18)	1.26 (0.79 to 2.01)	19	1.38 (0.69 to 2.76)	1.14 (0.56 to 2.32)
RBP-4										
Low <27.6 mg/L	357	62	1.00	1.00	46	1.00	1.00	18	1.00	1.00
Mid 27.6–35.0 mg/L	348	54	0.92 (0.63 to 1.32)	0.87 (0.60 to 1.27)	40	0.93 (0.61 to 1.42)	0.94 (0.60 to 1.46)	16	0.94 (0.48 to 1.84)	0.96 (0.48 to 1.89)
High >35.0 mg/L	355	60	0.99 (0.70 to 1.41)	0.86 (0.60 to 1.23)	30	0.67 (0.42 to 1.06)	0.69 (0.42 to 1.15)	12	0.68 (0.33 to 1.40)	0.67 (0.32 to 1.38)
Fetuin-A										
Low <236 mg/L	357	57	1.00	1.00	37	1.00	1.00	9	1.00	1.00
Mid 236–289 mg/L	348	52	0.93 (0.64 to 1.36)	0.93 (0.64 to 1.37)	37	1.05 (0.67 to 1.66)	1.05 (0.66 to 1.66)	19	2.21 (1.00 to 4.88)	2.41 (1.10 to 5.27)
High >289 mg/L	354	67	1.20 (0.84 to 1.71)	1.06 (0.74 to 1.52)	42	1.18 (0.76 to 1.84)	1.24 (0.78 to 1.96)	18	2.08 (0.93 to 4.64)	1.93 (0.87 to 4.28)
Uric acid										
Low <31 mg/L	369	61	1.00	1.00	43	1.00	1.00	15	1.00	1.00
Mid 31–36 mg/L	360	61	1.05 (0.74 to 1.49)	1.01 (0.71 to 1.44)	39	0.93 (0.60 to 1.43)	0.98 (0.63 to 1.52)	15	1.04 (0.51 to 2.13)	1.09 (0.50 to 2.38)
High >36 mg/L	382	62	0.99 (0.70 to 1.41)	0.88 (0.60 to 1.29)	45	1.04 (0.68 to 1.57)	1.08 (0.66 to 1.75)	20	1.31 (0.68 to 2.55)	1.42 (0.69 to 2.88)
Vitamin B6										
Low <33.1 nmol/L	346	56	1.00	1.00	39	1.00	1.00	14	1.00	1.00
Mid 33.1–48.8 nmol/L	353	60	1.06 (0.74 to 1.53)	1.09 (0.75 to 1.59)	40	1.05 (0.67 to 1.63)	1.06 (0.68 to 1.64)	20	1.47 (0.74 to 2.90)	1.62 (0.78 to 3.33)
High >48.8 nmol/L	355	57	0.99 (0.69 to 1.43)	1.00 (0.67 to 1.48)	34	0.88 (0.56 to 1.40)	0.91 (0.57 to 1.47)	15	1.09 (0.53 to 2.24)	1.27 (0.59 to 2.74)

*Adjusted for age only.

†Adjusted for age, smoking, diabetes, systolic blood pressure, total cholesterol, total triglycerides, body mass index and family history of premature CHD.

‡Significant test for trend in sub-hazard ratio (P<0.01).

BNP, B-type natriuretic peptide; CHD, coronary heart disease; CRP, C reactive protein; CVD, cardiovascular disease; GDF-15, growth differentiation factor-15; IHD, ischaemic heart disease; IL, interleukin; Lp-a, lipoprotein-a; MI, myocardial infarction; PAPP-A, pregnancy- associated plasma protein-A; RBP-4, retinol binding protein-4; VCAM-1, vascular cell adhesion molecule-1.

In the top third of the distributions of troponin and BNP, the highest values were for CHF (SHRs 3.37, 95% CI 1.39 to 8.14 and 3.23, 95% CI 1.45 to 7.23), respectively, adjusted for all conventional risk factors.

For acute MI/IHD, the SHR for troponin was only moderately elevated in the top third of the distribution (SHR 1.63, 95% CI 1.10 to 2.41). For stroke, the SHR for third of the distribution was not raised for troponin but was significantly raised for BNP (SHR 1.75 95% CI 1.06 to 2.88). Of the remaining biomarkers, only the lipids total cholesterol and Lp-a and the liver enzyme aspartate transaminase showed significant associations with either acute MI/IHD or stroke (for acute MI/IHD the top third SHR was 2.48 (95% CI 1.63 to 3.77) for total cholesterol and 1.84 (95% CI 1.26 to 2.69) for Lp-a; for aspartate transaminase, the respective SHRs were 0.68 (95% CI 0.46 to 0.99) for acute MI/IHD and 0.63 (95% CI 0.41 to 0.96) for stroke.

None of the significant associations between biomarkers and CVD risk showed non-linearity in the relationship. Tests of interaction with time showed no evidence that the strength of the significant associations changed with length of follow-up.

Table 3 provides a summary of the main results in which the adjusted SHRs have been calculated per third of each biomarker distribution (a summary figure across the distribution of each biomarker) for the individual CVD outcomes. Results significant at the 1% level of statistical significance are shown in bold type. Adjustment was made for the same conventional risk factors as listed for table 2.

**Table 3:** Sub-hazard ratios (95% CI) for MI/IHD, stroke and congestive heart failure per third of each biomarker distribution in a competing risk model for 1112 fasting men with no CVD at entry and complete covariate data

Biomarker	MI/IHD	Stroke	Congestive Heart failure
	Sub-hazard ratio (95% CI)	Sub-hazard ratio (95% CI)	Sub-hazard ratio (95% CI)
Troponin	**1.30 (1.07 to 1.59)**	0.83 (0.64 to 1.06)	**1.84 (1.21 to 2.80)**
BNP	1.10 (0.91 to 1.33)	1.33 (1.04 to 1.70)	**1.94 (1.26 to 2.97)**
CRP	1.10 (0.90 to 1.35)	1.01 (0.80 to 1.28)	1.00 (0.71 to 1.41)
IL-6	0.94 (0.78 to 1.14)	1.14 (0.91 to 1.42)	0.84 (0.57 to 1.22)
IL-6 receptor	1.02 (0.84 to 1.23)	0.92 (0.72 to 1.16)	1.04 (0.74 to 1.47)
Cholesterol	**1.55 (1.27 to 1.88)**	0.89 (0.72 to 1.10)	1.13 (0.78 to 1.63)
Triglyceride	1.08 (0.88 to 1.33)	1.01 (0.81 to 1.25)	0.77 (0.54 to 1.10)
Lp-a	**1.35 (1.12 to 1.63)**	0.90 (0.71 to 1.13)	0.69 (0.45 to 1.04)
VCAM-1	0.90 (0.75 to 1.10)	1.05 (0.84 to 1.32)	1.28 (0.88 to 1.86)
E-selectin	0.88 (0.73 to 1.07)	1.17 (0.93 to 1.47)	1.16 (0.79 to 1.69)
Alkaline phosphatase	0.92 (0.76 to 1.11)	1.22 (0.98 to 1.51)	0.92 (0.65 to 1.33)
Aspartate transaminase	0.83 (0.69 to 1.00)	0.77 (0.61 to 0.98)	1.31 (0.91 to 1.84)
Alanine transaminase	0.96 (0.79 to 1.18)	0.99 (0.79 to 1.25)	1.00 (0.70 to 1.43)
Gamma-GT	0.89 (0.73 to 1.09)	1.00 (0.79 to 1.26)	0.98 (0.71 to 1.34)
Glutamate dehydrogenase	1.00 (0.78 to 1.32)	1.07 (0.77 to 1.49)	1.11 (0.66 to 1.85)
White cell count	1.20 (1.00 to 1.45)	1.20 (0.93 to 1.53)	1.13 (0.75 to 1.70)
Viscosity	1.12 (0.93 to 1.35)	1.13 (0.90 to 1.41)	1.21 (0.84 to 1.75)
Fibrinogen	1.16 (0.96 to 1.39)	1.11 (0.89 to 1.37)	0.95 (0.65 to 1.38)
Ferritin	0.94 (0.79 to 1.13)	1.02 (0.81 to 1.28)	1.33 (0.88 to 2.01)
GDF-15	0.95 (0.77 to 1.17)	0.94 (0.74 to 1.21)	1.08 (0.71 to 1.66)
PAPP-A	1.02 (0.84 to 1.23)	1.14 (0.91 to 1.44)	1.41 (1.00 to 1.97)
Cystatin	1.01 (0.83 to 1.23)	1.14 (0.90 to 1.45)	1.01 (0.66 to 1.53)
Creatinine	0.85 (0.70 to 1.02)	0.89 (0.71 to 1.12)	1.21 (0.80 to 1.82)
Glucose	1.09 (0.88 to 1.34)	1.12 (0.88 to 1.42)	1.07 (0.76 to 1.49)
RBP-4	0.93 (0.77 to 1.11)	0.84 (0.66 to 1.07)	0.82 (0.58 to 1.17)
Fetuin-A	1.03 (0.86 to 1.24)	1.11 (0.88 to 1.41)	1.31 (0.94 to 1.82)
Uric acid	0.94 (0.78 to 1.13)	1.04 (0.81 to 1.33)	1.19 (0.83 to 1.71)
Vitamin B6	1.00 (0.82 to 1.21)	0.96 (0.76 to 1.21)	1.12 (0.79 to 1.57)

Results in bold are significant (P<0.01).

Adjusted for covariates: age, smoking, diabetes, systolic blood pressure, total cholesterol, total triglycerides, body mass index and family history of premature CHD.

BNP, B-type natriuretic peptide; CHD, coronary heart disease; CRP, C reactive protein; CVD, cardiovascular disease; GDF-15, growth differentiation factor-15; IHD, ischaemic heart disease; IL, interleukin; Lp-a, lipoprotein-a; MI, myocardial infarction; PAPP-A, pregnancy- associated plasma protein-A; RBP-4, retinol binding protein-4; VCAM-1, vascular cell adhesion molecule-1.

SHRs for non-ischaemic CHF showed strong significant associations with troponin and BNP, while lesser associations were shown for troponin in the case of MI/IHD events and for BNP in the case of stroke events. Total cholesterol and Lp-a showed associations for MI/IHD events only (with SHRs larger than that for troponin). No other biomarker showed a statistically significant SHR with each of these individual CVD outcomes in these men with no evidence of prior CVD.

Thus, for CHF, there are strong associations with troponin and BNP and weaker associations between these biomarkers and MI/IHD and stroke. The strongest associations for MI/IHD were with total cholesterol and Lp-a.

For the CHF outcome, we tested the effect of adjusting for all conventional risk factors and troponin and BNP simultaneously; the SHR for troponin was slightly reduced (from 1.84 to 1.64) with no change for BNP. For MI/IHD, we examined the effect of adjusting for total cholesterol and Lp-a together with conventional risk factors (other than total cholesterol). The SHR for total cholesterol was slightly reduced (1.55 to 1.48), but the SHR for Lp-a was unchanged.

Similarly, we calculated Harrell’s C statistic with and without the inclusion of both troponin and BNP. Using the competing risks model for MI/IHD including the conventional risk factors and Lp(a), the inclusion of troponin after the addition of BNP increased the value of the C statistic from 0.678 to 0.688, whereas the addition of BNP to a model that included troponin produced only a modest change from 0.685 to 0.686. For CHF, both troponin and BNP appeared to contribute to risk prediction: the addition of troponin to the model that included the conentional risk factors along with BNP increased the C statistic from 0.767 to 0.777, while the addition of BNP to the troponin model increased the C statistic from 0.745 to 0.775. However, none of these changes attained statistical significance (online supplementary table 2), possibly because the number of outcome events, particularly for CHF, was small. C statistics were not calculated for stroke since none of the biomarkers was significant for this endpoint.

## Discussion

In this report, we have examined the relative contributions of a panel of biomarkers to the prediction of three major cardiovascular events, fatal and non-fatal, in a general population of men aged 55–69 years in the early 1990s. During an average follow-up period of 13 years, many men experienced more than one type of cardiovascular event, but acute MI/IHD events predominated, followed by stroke and (chronic) CHF (figure 1A). Acute heart failure associated with acute MI/IHD was excluded from the CHF events, as defined in our study. As the general population included men with a prior history of CVD or clinical evidence of this from a resting ECG, we defined a subcohort that excluded these men (figure 1B). Among the 458 men in this subcohort who experienced a first event, acute MI/IHD events predominated (50%) with fewer strokes (36%) and CHF events (14%). These CHF events were categorised by the authors as non-ischaemic in origin.^24^ However, in the full cohort, it was clear that prior CVD preceded chronic CHF in a high proportion of cases; some 55% having a history of MI or stroke prior to the baseline examination at phase 3, symptoms suggestive of IHD or resting ECG ischaemia without symptoms. The latter comprised a group of men without symptoms of CVD at baseline and without a prior CVD event. Therefore, a significant number of men who went on to develop CHF would have been unrecognised as being at risk at baseline.

A major objective of our study was to examine the predictive value of our panel of 28 biomarkers for acute MI/IHD, stroke and non-ischaemic CHF. Following adjustment for all classical risk factors, high-sensitivity troponin and the natriuretic peptide BNP showed the strongest predictive values for non-ischaemic CHF, and troponin showed a weaker association with acute MI/IHD as previously noted for CHF (not necessarily defined as non-ischaemic) in the Atherosclerosis Risk in Communities (ARIC) and Cardiovascular Health Study cohorts.^25 26^ Furthermore, we found that both BNP and troponin contributed independently to the risk of CHF but only troponin and, more strongly, total cholesterol and Lp-a (again acting largely independently), predicted risk of acute MI/IHD. Nambi *et al*^27^ from the ARIC study reported that troponin and BNP together have significant value in the prediction of subsequent CHF, which was confirmed in our present report in subjects without prior CVD. Unsurprisingly, we found that baseline levels of BNP predicted stroke risk but not acute MI/IHD.

In a major trial of more than 17 000 men and women with low-density lipoprotein cholesterol levels less than 3.4 mmol/L, baseline levels of high sensitivity troponin in the highest tertile were associated with a HR of 2.2 (95% CI 1.6 to 3.1) for a first CVD event and 2.6 (95% CI 1.8 to 3.8) for all-cause mortality.^28^ Comparable figures for BNP were 1.9 (95% CI 1.4 to 2.7) for CVD and 1.5 (95% CI 1.0 to 2.0) for all-cause mortality. In a previous report, we found that troponin and BNP were strongly predictive of CVD mortality but not for non-CVD mortality, and we suggested that biomarkers should be examined for specific causes of mortality in cohort studies.^15^ With the exception of total cholesterol and Lp-a and fatal and non-fatal MI/IHD, none of the other panel of biomarkers, including several inflammatory markers, showed convincing associations with the individual cardiovascular outcomes in this subcohort of men. Although previous cohort studies^13^ and reviews^29 30^ have suggested a role for inflammatory biomarkers in the pathogenesis of CHF, we found no evidence of this in our long-term follow-up study of non-ischaemic CHF events. Differences in the definition of CHF events and the length of follow-up may account for these differences.

### Strengths and limitations

The major strength of our study is that we have defined first events for acute MI/IHD, stroke and CHF in subjects without a history of symptomatic or clinical (ECG) evidence of prior CVD. Measurement of troponin, and more particularly of BNP, may be useful as a screening test in the middle-aged population of men to detect those at higher risk of developing subsequent CHF. Limitations of our report are that the number of first events of CHF in our subcohort is small, statistical power is modest and our results are restricted to a specific population of men. We cannot exclude the possibility that ischaemia that did not result in clinical symptoms may have occurred during the follow-up period. However, in a sensitivity analysis, which excluded men with ECG evidence of ischaemia and atrial fibrillation at baseline resulting in a subcohort of men totalling 1642, SHRs for the top third of the distributions of troponin were reduced for CHF and acute MI/IHD outcomes: 1.98 (95% CI 1.02 to 3.98) and 1.40 (95% CI 1.00 to 1.97). Similarly, the SHR for the top third of the distribution of BNP was reduced in the case of CHF events: 2.43 (95% CI 1.27 to 4.66). This suggests that men with a history of severe chest pain and those with symptoms of angina of effort also have myocardial damage or ischaemia without evidence of this on a resting ECG. We conclude that:

- In this cohort of men of average age 62 years followed for an average of 13 years acute MI/IHD events occurred twice as commonly as CHF events, with stroke events taking an intermediate rank.
- In a subcohort of men without prior CVD, 55% of first CHF events during follow-up were not preceded by other cardiovascular outcomes or occurred as the only type of outcome.
- Men without prior CVD in the top third of the distributions of troponin and BNP at baseline examination had a greater than threefold risk of experiencing a CHF event compared with men in the bottom third in contrast to 1.63 tmies therisk of MI for troponin and 1.75 times the risk of stroke for BNP.
- Troponin and BNP could be considered as potentially useful screening tools to detect subjects without prior CVD at increased risk of developing CHF in subsequent years in addition to having lesser roles for predicting subsequent risk of MI for troponin and stroke for BNP.
