# Supplementary material for: Troponin and BNP are markers for subsequent non-ischaemic congestive heart failure: the Caerphilly Prospective Study (CaPS)
Source: Open Heart. 2018 Feb 23;5(1):e000692. doi: 10.1136/openhrt-2017-000692 (PMC5845403; doi:10.1136/openhrt-2017-000692)
Supplement: Supplementary file 1 [file openhrt-2017-000692supp001.pdf]

Supplementary tables

Supplementary Table 1 Sample sizes used in this report

Total number of men examined	2171
Fasting for blood sample	1911
No previous CVD	1279
Fasting & No previous CVD	1176
Fasting, No previous CVD and complete covariates	1112

Supplementary Table 2 Comparing the predictive power of survival models using the increment in Harrell's C statistic resulting from the addition of biomarkers to the model

		Before adding			After adding			
**End Point**	**Biomarkers in model**	**C statistic**	***95%***	***CI***	**C statistic**	***95%***	***CI***	**P**
MI/IHD	Troponin added to BNP	0.678	*0.637*	*0.720*	0.688	*0.626*	*0.708*	0.15
MI/IHD	BNP added to Troponin	0.685	*0.644*	*0.727*	0.686	*0.644*	*0.728*	0.45
CHF	Troponin added to BNP	0.767	*0.694*	*0.839*	0.777	*0.706*	*0.849*	0.54
CHF	BNP added to Troponin	0.745	*0.670*	*0.820*	0.775	*0.704*	*0.846*	0.16

All modelsincluded the following covariates: age, smoking, diabetes, systolic blood pressure, total cholesterol, total triglycerides, body mass index and male/female family history of premature CHD. The MI/IHD model also included Lp(a). The Troponin and BNP values were fitted to the model as thirds.
